# Review of Toxoplasmosis: What We Still Need to Do

**DOI:** 10.3390/vetsci12080772

**Published:** 2025-08-18

**Authors:** Muhammad Farhab, Muhammad Waqar Aziz, Aftab Shaukat, Ming-Xing Cao, Zhaofeng Hou, Si-Yang Huang, Ling Li, Yu-Guo Yuan

**Affiliations:** 1 College of Veterinary Medicine, Yangzhou University, Yangzhou 225009, China; farhab.dvm@gmail.com (M.F.); caomingxing@stu.swun.edu.cn (M.-X.C.); zfhou@yzu.edu.cn (Z.H.); siyang.huang@hotmail.com (S.-Y.H.); ll13665200796@163.com (L.L.); 2 Jiangsu Co-Innovation Center of Prevention and Control of Important Animal Infectious Diseases and Zoonoses, Yangzhou University, Yangzhou 225009, China; 3 Institute of Microbiology, University of Veterinary and Animal Sciences, Lahore 54600, Pakistan; waqar.aziz@uvas.edu.pk; 4 College of Veterinary Medicine, South China Agricultural University, Guangzhou 510642, China; 5 College of Veterinary Medicine, Shanxi Agricultural University, Taiyuan 030800, China

**Keywords:** *Toxoplasma gondii*, lifecycle, diagnosis, treatment, control

## Abstract

*Toxoplasma gondii* is responsible for the disease toxoplasmosis and has the broadest host range among apicomplexan parasites, as it infects virtually all warm-blooded vertebrates. Toxoplasmosis is a zoonotic and emerging public health concern with considerable morbidity and mortality, especially in the developing world, affecting approximately one-third of the world’s human population. Clinical presentation varies among species, and the infection establishes lifelong chronicity in hosts. Most of the host species (including healthy humans) are asymptomatic on the one hand, it is fatal to marsupials, neotropical primates and some marine mammals on the other hand. In immunocompetent humans, infection is typically asymptomatic, whereas immunocompromised individuals may develop disseminated disease affecting virtually any organ system—most commonly reproductive, cerebral, and ocular systems. Toxoplasmosis spreads by ingestion of food or water contaminated with *T. gondii* oocysts, consumption of undercooked/raw meat containing tissue cysts, transplacental transmission from mother to fetus, or by receiving infected organ/blood from the infected individual. Toxoplasmosis is mainly diagnosed by serologic tests and polymerase chain reaction (PCR). It is treated with pyrimethamine combined with sulfadiazine or clindamycin, often supplemented with leucovorin, atovaquone, and dexamethasone. Despite having many potent anti-*T. gondii* antigenic candidates, there is no commercially available vaccine for humans due to many factors, including the complex life cycle of the parasite and its evasion strategies. To date, the only commercially available anti-*T. gondii* vaccine is for sheep, licensed for veterinary use to prevent ovine abortions. In this review, we have summarized the current understanding of toxoplasmosis.

## Simple Summary

*Toxoplasma gondii* is the causative agent of toxoplasmosis, a zoonotic disease infecting nearly one-third of humans worldwide. Although often asymptomatic in healthy individuals, it poses serious risks to immunocompromised patients, pregnant women, and certain animal species, leading to severe neurological, ocular, or fetal complications. Transmission occurs through contaminated food/water (oocysts), undercooked meat (tissue cysts), or congenital/transplant routes. Diagnosis relies on serology and PCR, with treatment (e.g., pyrimethamine-sulfadiazine) reserved for high-risk cases. Despite promising antigen candidates, no human vaccine exists due to the parasite’s complex biology; the only licensed vaccine prevents abortions in sheep. This review highlights current knowledge on toxoplasmosis, emphasizing its global health impact and unmet medical needs.

## 1. Introduction

The Greek word “*Toxoplasma*” means “arc-shaped body”, and was named by Nicolle and Manceaux for its arc-shaped tachyzoites [1]. It is an obligate intracellular protozoan that causes toxoplasmosis in warm-blooded animals (including humans, other mammals, and birds) worldwide. Although it can be isolated from cold-blooded animals, it does not parasitize them [2,3,4]. Unlike closely related parasites, which typically have narrow host ranges, *Toxoplasma gondii* has a remarkably broad distribution. Felids (family *Felidae*) are the only definitive hosts, and sexual reproduction occurs in their small intestine, resulting in the excretion of oocysts into the environment **[2]**. *Toxoplasma gondii* has medical and veterinary importance and is used as a model for apicomplexan organisms. *Toxoplasma gondii* can be grown and maintained in virtually any warm-blooded cell line and in mice. Additionally, *T. gondii* is readily amenable to genetic manipulation and has high transfection efficiency [5]. Even though most species and immunocompetent individuals are immune to this infection, the fact that it can infect virtually all mammals has gained increasing attention as an excellent model system to study host–pathogen interactions [6,7]. It infects up to one-third of the world’s human population and is the second most common asymptomatic intrauterine infection worldwide [7].

Over the past decades, there have been several breakthroughs in human and animal toxoplasmosis. The objective of this review is to concisely summarize the current trends in human and animal toxoplasmosis with special emphasis on the epidemiology of the disease, including its global distribution, pathophysiology, clinical presentations, diagnosis, treatment, and preventive measures. It also aims to provide an overview of the present understanding of *T. gondii* vaccine development and the challenges that are limiting its commercial availability for human use. As far as molecular epidemiology is concerned, we have considered geographical mapping. *Toxoplasma gondii* PopSet sequences were accessed on 6 June 2025 [8]. From the extensive literature on toxoplasmosis treatment, we drafted a summary of toxoplasmosis. The treatment regimens were specially emphasized on drug selection rather than drug doses, dose intervals, mechanism of action, etc.

## 2. History

### 2.1. Etiology

The name “*Toxoplasma gondii*” was proposed based on its morphology and the host *Ctenodactylus gundi* (originally misidentified as *C. gondi*) [9]. In 1937, *T. gondii* was isolated from guinea pig brains and maintained in the laboratory through serial mouse passage [10]. In 1939, *T. gondii* was isolated from a fatal case of human infantile encephalitis [11]. This isolate, later designated the RH strain, became the prototype for studying acute toxoplasmosis due to its consistent lethality in laboratory animals. In 1941, *T. gondii* isolates from humans and animals were established as the same species. In 1992, *T. gondii* was classified based on infection in mice as Type I, II, III, and atypical. The *T. gondii* genome was mapped in 2005 [12]. Recent studies reported that the *T. gondii* Regulator of Cystogenesis 1 gene is required for the formation of *T. gondii* bradyzoites [9,12,13].

### 2.2. Historical Background of the Disease in Animals

*Toxoplasma gondii* was first reported in 1908 in tissue of *Ctenodactylus gundi* in Tunisia. In 1910 in Italy, there was the first report of fatal toxoplasmosis in a dog that died of acute visceral toxoplasmosis. The first case of toxoplasmosis in a cat was reported in 1942 in the Middletown, New York, USA. Toxoplasmosis in sheep was discovered in 1957 from aborted fetuses. Cattle and horses are resistant to clinical *T. gondii*, and there is no confirmed case of clinical toxoplasmosis in either of these animals. Cattle are considered resistant to clinical toxoplasmosis and viable *T. gondii* is rarely isolated from bovine tissues.

In 1954, Hartley et al. [14] observed ovine abortions and isolated *T. gondii*-like bodies from fetal membrane lesions. They termed these “New Zealand type II abortions”, but cautioned that attributing causality to *T. gondii* was premature. In 1957, Buxton [15] inoculated sheep with gauze-filtered suspension of fetal cotyledons from the field cases of *T. gondii*-associated abortion, and then they successfully isolated *T. gondii* from these inoculated sheep. They proved that the cause of New Zealand Type II Abortion is toxoplasmosis [14,15].

*T. gondii* was isolated for the first time from sea otters in 2000 [9,12,13]. In 2012, Dubey et al. [16] reported the first confirmed case of acute toxoplasmosis in cattle, which died of acute toxoplasmosis in New Zealand. Diagnosis was confirmed by presence of numerous tissue cysts, which was confirmed by immunohistochemical staining with bradyzoite-specific *T. gondii* antibodies (*BAG1*) [16].

Kimble et al. [17] presented the first report of clinical toxoplasmosis in horses. An adult American Quarter Horse gelding with a history of weight loss presented with an acute onset of colic, fever, soft faeces, and elevated liver enzymes. Within and surrounding necrotic areas were free and intrahistiocytic clusters of protozoal tachyzoites. Similar but milder inflammation was evident in the spleen, lungs, and liver. Necrotizing typhlitis was also evident. Immunolabelling for *T. gondii* was positive and the ultrastructural morphology of the protozoa was compatible with *T. gondii* [17,18].

### 2.3. Parasite Morphology and Life Cycle

In 1960, it was discovered that cystic organisms (later termed bradyzoites) resist pepsin and trypsin digestion [19]. In 1970, oocysts were discovered, and both asexual and sexual stages were identified in intestine of cats. In 1972, the asexual enteroepithelial stages were designated as types A–E. The term “bradyzoite”, also called cystozoites, was proposed in 1973 for the cystic organisms. In 1976, it was found that inoculation of tachyzoites (alternatively called trophozoite, feeding form, proliferative form, endodyozoite) to mice leads to tissue cysts formation as early as 3 days, and cats can shed oocysts for up to 18 days from the day of infection. A summary of milestones of *T. gondii* in animals is presented in Table 1.

### 2.4. Notable Outbreaks/Epizootics in Domestic and Wild Species

In 1954, New Zealand experienced lamb abortion rates of 15-20%, initially suspected to result from toxoplasmosis [14]. Although Hartley et al. [14] successfully isolated *T. gondii* from the samples of aborted samples, they were not sure that *T. gondii* was the actual cause until it was confirmed by Buxton [15], by experimentally infecting sheep in 1957. During the lambing season of 2005, a toxoplasmosis abortion storm occurred in a flock of purebred Suffolk ewes on a farm in Texas, USA. Only 14 healthy lambs were born, and 38 abortuses, mummies, and weak or stillborn lambs were delivered. Another 15 fetuses identified by ultrasound were presumably resorbed or were aborted undetected. In the 2006 lambing season, two of 26 ewes delivered *T. gondii-infected* lambs. *Toxoplasma gondii* was isolated from the brain and heart of a lamb from the second ewe. In subsequent seasons, the ewes lambed normally. The results of the present study support the hypothesis that most sheep that have aborted due to *T. gondii* develop protection against future toxoplasmosis-induced abortion, but the protection is not absolute [83]. From 2015 to 2017, investigations identified 22 ovine abortion outbreaks in 20 Spanish farms, and viable *T. gondii* were isolated from 11 of these 22 outbreaks [84]. Representative samples were collected from abortion outbreaks (*n* = 31) and from chronically infected adult sheep at slaughterhouses (*n* = 50) in different Spanish regions, and bioassayed in mice. As a result, 30 isolates were obtained: 10 from abortion-derived samples and 20 from adult myocardial tissues [84]. Gabardo et al. [85] described an outbreak of *T. gondii* in a sheep herd in 2010 in Brazil. A total of 40 out of 100 pregnant ewes were aborted in the last month of pregnancy or had stillbirths. The fetal central nervous system has intralesional cysts positive for *T. gondii* by immunohistochemistry [85]. Recently, a study in Spain described two outbreaks of abortions, one affecting a dairy flock (abortions in 30 of 239 pregnant ewes) and the other affecting a meat flock, abortions in 70 of 210 pregnant ewes [86]. In 1979 [76] reported congenital toxoplasmosis in three flocks of sheep from Tasmania as follows. (i) In December 1973, three does presented premature parturition or stillbirth, and their kids were seropositive for *T. gondii* through IFAT at 1:16 dilution. No significant reactions were obtained in brucellosis and leptospirosis agglutination tests. (ii) In 1976, there was a case of many abortions or stillbirths and in October–November 1977, five stillbirths from the same flock. Dam was *T. gondii* IFAT positive with titre of 1:512. (iii) In 1978, abortion in an Angora goat resulted in twin kids. *Toxoplasma gondii* from one of these kids was successfully isolated through a mouse bioassay.

In 2001, abortions and stillbirths were seen in a pig farm from Jiangxi Province; 42.0% of pigs were affected with 8.0% mortality. *Toxoplasma gondii* tachyzoites were isolated from many organs under a light microscope, and from mice inoculated with tissues of dead pigs [5]. In another outbreak from a farm in Guangzhou city, sows in late pregnancy aborted. A total of 33.0% of sows were affected with 2.0% mortality. *Toxoplasma gondii* was recovered from mice inoculated with tissues of a dead sow [5]. In 2004, 19 pigs from a farm containing 260 sows and 960 fattening pigs in Jinchang, Gansu Province, China died. Tachyzoites of *T. gondii* were found in body fluids of mice inoculated intraperitoneally with ground samples from the heart, liver, spleen, and brain of two sick pigs. In addition, the inoculation of five pigs with *T. gondii* tachyzoites caused death in two of the pigs [87]. During October 1994, there were four outbreaks of disease with mortality in different pig herds in two different provinces, respectively, Lombardia and Emilia-Romagna regions (North Italy). The morbidity was high (50–60%), and mortality ranged from 10% to 42% [88].

Hector’s dolphins (*Cephalorhynchus hectori*), a small, endangered coastal species, are endemic to New Zealand. In 2013, Roe et al. [89] found that 7 of 28 (25%) dolphins died due to disseminated toxoplasmosis, including 2 of 3 Maui’s dolphins, a critically endangered sub-species [89]. *Toxoplasma gondii* infection was detected by Dubey et al. [90] in bottlenose dolphins (*Tursiops truncatus*) from a sea aquarium in Canada in 2009. All of them were seropositive; among them, two dolphins died. *Toxoplasma gondii* tissue cysts were identified in histological sections of the brain of one dolphin. Another dolphin did not have visible organisms, but viable *T. gondii* was isolated by bioassay in mice and cats from its brain and skeletal muscle [90].

During 2001–2015, 14 out of 183 seals diagnosed to be infected by protozoal infections by histopathology, of which (*n* = 8) were confirmed cases of *T. gondii*-related mortality [91], including the one described by [14]. *Toxoplasma gondii* was associated with two cases of suspected cases among these marine mammals [91].

In 1964, McAllister et al. [92] reported an outbreak of toxoplasmosis that killed 44 of a herd of 56 chinchillas and produced four abortions with the loss of a total of five fetuses. A wild mouse thought to be the house mouse (*Mus musculus*), caught in a trap in the basement housing the chinchillas, was found to be infected with *T. gondii* [92].

An epizootic of toxoplasmosis occurred among 22 adult and 30 kit black-footed ferrets (*Mustela nigripes*) maintained under quarantine conditions at the Louisville Zoological Garden (Kentucky, USA) in 1992. Two adults and six kits died with acute disease. Toxoplasmosis was confirmed by immunohistochemical staining. Chronic toxoplasmosis resulted in the death of an additional 13 adult ferrets [93].

Between 2006 and 2010, Budapest Zoo and Botanical Garden of Hungary faced a fatal toxoplasmosis outbreak in Tammar wallabies (*Macropus eugenii*), which led to the death of six animals. *Toxoplasma gondii* was confirmed through immunohistochemical examination. In another four specimens, histopathology revealed *T. gondii*-like organisms (which could not be differentiated from *Neospora caninum* solely by morphology), and in another 11 animals, toxoplasmosis was the possible cause of death. The current zoo population of 12 Tammar wallabies was tested for *T. gondii* IgG antibodies by the modified agglutination test (MAT), with negative results [94].

In 2017, Guthrie et al. [95] described an outbreak of fatal toxoplasmosis in nine red-necked wallabies (*Macropus rufogriseus*) (RNW) [95]. In 2018, Verma et al. [96] isolated *T. gondii* from brains and/or muscles of 22 of 44 northern sea otters (*Enhydra lutris kenyoni*) from Washington State, USA. The isolates were bioassayed in mice and viable *T. gondii* were isolated [96]. In the spring of 1999, a large Wisconsin mink (*Mustela vison*) farm experienced an outbreak of abortions, stillborn kits, and kit mortality. A total of 7,800/1,976 females lost their entire litter either from abortion or neonatal mortality, respectively. Overall kit mortality was 10,408. Toxoplasmosis was diagnosed by the detection of *T. gondii* tachyzoites by immunohistochemistry [97]. 

In 1999, Bouer reported fatal outbreak of toxoplasmosis in a Zoo in Brazil in three wooly monkeys (*Lagothrix lagotricha*) [98]. Carme et al. [99] reported outbreaks of toxoplasmosis in two episodes in 2001 and 2006 in a captive breeding colony of squirrel monkeys (*Saimiri sciureus*) in the Institut Pasteur in French Guiana. During both outbreaks, a total of 50 monkeys died and none recovered spontaneously [99]. In 2019, four squirrel monkeys (*Saimiri* sp.) died in Japan, followed by seven of thirteen dying in South Korea in 2018.

In another outbreak seven howler (*Alouatta* sp.) monkeys died of disseminated acute toxoplasmosis during 34 days, with intervals of 2–15 days between deaths from Brazil. These monkeys were kept in two side-by-side enclosures, suggesting in-contact transmission [100].

Seven of 30 canaries in an aviary in New Zealand developed ophthalmic problems. Clinically, five birds had unilateral and two birds had bilateral lesions characterized by conjunctivitis, crusty exudates on eyelids, and collapse of the eyeball. Numerous *T. gondii* tachyzoites were seen in the detached retina and vitreous humor of acutely affected birds. The diagnosis of toxoplasmosis was confirmed by immunohistochemical staining with *T. gondii* antiserum [101]. Vielmo et al. [102] described a toxoplasmosis outbreak in domestic chickens and guinea fowl in southern Brazil. 22 birds (13 chickens and 9 guinea fowl) from a total of 76 birds (47 domestic chickens and 29 guinea fowl), showed clinical signs of lethargy, anorexia, and neurological signs, and 15 (9 chickens, 6 guinea fowl) died within 24–72 h. Histopathological findings included tissue necrosis with intralesional *T. gondii*. Immunohistochemistry for *T. gondii* was positive [102]. In 2004, five lories died of acute toxoplasmosis in an aviary in South Carolina, United States. For the isolation of *T. gondii*, tissues were bioassayed in mice and viable *T. gondii* was isolated from all five of five lories [49]. In 2008, there was a report of death of three (out of ten) Nicobar pigeons (*Caloenas nicobaria*) in an aviary collection in South Africa, without any clinical symptoms. Numerous protozoal tachyzoites were present in all organs and there was strong positive immunohistochemical (IHC) labelling of these organisms for *T. gondii* [59]. In 2011, three 13-month-old black-footed penguin chicks (*Spheniscus demersus*) died from acute toxoplasmosis within 24 h of showing central nervous signs [61]. In the middle of June 1952, Erichsen et al. [103] reported an epidemic outbreak of toxoplasmosis in a chicken flock in south-eastern Norway. There was an unusually high mortality among chickens on a farm, without showing signs of illness prior to death. During a period of three months further two dead chickens were diagnosed from the affected farms. From these animals *T. gondii* was successfully isolated, and it was decided to eradicate the whole flock [103].

A severe outbreak of toxoplasmosis was observed in a hare ranch (*Lepus timidus ainu*) in Sapporo, Japan. Eight hares out of thirteen had revealed the symptoms and all died. The adult hare suffered in a very high percentage (seven out of eight), compared with that of the young (one out of five). Parasites were demonstrated heavily in the liver, spleen, and mesenteric lymph node by the Giemsa-stained smear. The isolation of the parasites was successful in one case after four successive inoculations to mice [104]. Notable outbreaks/epizootics of toxoplasmosis in domestic and wild species are presented in Table 2.

### 2.5. Transmission

Congenital *T. gondii* infection in humans was described in 1939. In 1959, it was found that congenital infections in mice can produce infected offspring for at least 10 generations. In 1965, it was discovered that *T. gondii* infectivity is associated with cat feces. In 1970, the sexual phase of the parasite in the small intestine of the cat was discovered [24]. The schizogony and gametogony were identified in cat enterocytes and characterized morphologically and biologically. Pigs and mice (and presumably humans) can be infected by ingesting even 1 oocyst, whereas 100 oocysts may not infect cats. Cats can shed millions of oocysts after ingesting only 1 bradyzoite, while ingestion of 100 bradyzoites may not infect mice orally [12].

### 2.6. Human Toxoplasmosis

The chronology of *T. gondii* transmission routes was established through key discoveries: congenital infection (1939), carnivorism (1960), and feco-oral transmission (1965) [11].

First report of acquired toxoplasmosis was from a 6-year-old boy in the USA in 1941, and this isolate was given the child’s initials (RH) and became the famous RH strain. *Toxoplasma gondii* was isolated from the heart, spleen, blood, and other tissues in 1940–1941. In 1956, it was reported that lymphadenopathy was a frequent sign of acquired toxoplasmosis. Acute toxoplasmosis-induced encephalitis was reported in 1983, in which almost all cases resulted from reactivation of chronic infection initiated by the immune suppression due to HIV infection. In 1988, the first postnatal acquired toxoplasmosis presenting ocular disease (retinochoidal scars) was reported.

In 1974, results of the 15-year study demonstrated that (1) early pregnancy infections are more damaging to the fetus; (2) not all maternal infections transmit vertically; (3) women seropositive before pregnancy did not transmit infection to the fetus; and (4) spiramycin reduces transmission but not severity of disease in infants [12] (Table 3).

### 2.7. Diagnosis, Treatment, and Control

Diagnostic advances began with development of the first serologic anti-*T. gondii* diagnostic test called the Sabin–Feldman dye test in 1948, which became the gold standard for detecting anti-*T. gondii* antibodies across species owing to its high sensitivity [30], followed by the development of many antibody-based diagnostics. Detection of *T. gondii* DNA was achieved, targeting the tachyzoite *B1* gene, in 1989 through PCR. In 1941, sulfonamides were reported to be effective against murine toxoplasmosis [31]. In 1958, spiramycin was found to have antitoxoplasmic activity in mice. In 1973, clindamycin was documented as an alternative to sulfonamides [34]. In the 1990s, it was concluded that excluding cats from pig facilities can reduce *T. gondii* infection in pigs. Vaccination of sheep with a live cyst-less strain of *T. gondii* reduces neonatal mortality in lambs [36].

## 3. Life Cycle and Immunology/Immunopathogenesis

### 3.1. Life Cycle in Definitive Host (Felids)

Sexual reproduction of *T. gondii* is restricted to felids because their intestines lack delta-6-desaturase, creating a linoleic acid-rich environment that supports parasite development [26,107,108].

Bradyzoites in tissue cysts are the primary infectious form for both feline oocyst production and natural human infection, because they are more acid-resistant [109] and they can survive up to 2 h under gastric conditions. The evolution of bradyzoites is one of the most important strategies adopted by *T. gondii* to survive within the gastric proteolytic environment [19]. The presence of bradyzoites has been detected even during acute infections, suggesting rapid stage conversion occurs post-infection [110].

Sporozoites may convert to tachyzoites, and tachyzoites may convert to bradyzoite tissue cysts, and when tissue cysts rupture, a few bradyzoites return to the intestinal epithelium to initiate the enteroepithelial cycle; this event is unpredictable. Enteroepithelial stages preceding the formation of gamonts have not been found [111,112,113]. The prepatent period for oocyst excretion exceeds 18 days after ingesting sporulated oocysts. Unsporulated oocysts are nonpathogenic orally to cats [114].

After ingestion, most of the tachyzoites are destroyed by the gastric acidic environment. Pharyngeal-buccal mucosal invasion may occur [110,115], which can lead to excretion of *T. gondii* oocysts with a prepatent period of 18 days or more, though this occurs less efficiently than with bradyzoite ingestion.

#### 3.1.1. Motility, Invasion, and Egress

*Toxoplasma gondii* zoites move along a substrate by a gliding process that maintains anteroposterior polarity, maintaining their crescent shape. This locomotion reaches speeds of up to 10 μm/s in vitro [116]. This gliding motility consists of irregular corkscrew-like trajectories [117]. *Toxoplasma gondii* glide, invade, and egress through an actin-myosin-based motility machinery called the glideosome, which is a multi-protein complex anchored to *T. gondii*’s inner membrane complex (IMC), comprising Myosin A, actin, Myosin Light Chain 1, Gliding-Associated Proteins, surface adhesins [118,119]. The essential force comes from the myosin motor TgMyoA, which binds to TgACT1 (actin) and propels it unidirectionally toward the parasite’s rear. For attachment to host cells, surface adhesins like TgMIC2 and TgAMA1 engage host receptors, creating traction points. The glideosome then translocates these adhesins rearward, pulling the parasite forward in a substrate-dependent manner [120]. This (active) invasion is completed in less than 20 s, and during invasion, the same machinery drives the parasite into the host, forming a parasitophorous vacuole. The glideosome also facilitates egress by reactivating the actin–myosin motor, to rupture the host cell membrane (lytic egress) to disseminate and infect new cells [120,121,122].

#### 3.1.2. Schizogony

Bradyzoites initiate schizogony upon invading feline intestinal epithelial cells, an asexual reproduction process in which these zoites undergo repeated nuclear divisions without immediate cytokinesis, forming multinucleated schizonts [111]. These schizonts subsequently undergo cytokinesis, producing merozoites. Collectively, this developmental progression from bradyzoites/sporozoites to merozoites via schizont intermediates defines schizogony in *T. gondii* [123]. The enteroepithelial stages consist of five types, named as Type A, B, C, D, and E and gamonts [114]. All five types are enteroepithelial, but only Types B-E are schizonts, implying Type A to be an earlier stage. Although type B meets schizont criteria, schizogony has not been observed in Types A and B, and only Types C, D, and E undergo schizogony [26]. *Toxoplasma gondii* schizonts and merozoites are not infective to mice. Type B and C schizonts develop in both intestinal enterocytes and lamina propria, while Types D and E are restricted to enterocytes [124].

#### 3.1.3. Gametogenesis

The sexual cycle starts 2 days after ingestion of tissue cysts by the cat. The gametes originate from gametocytes formed during gametogony, which is initiated by merozoites (formed from types D/E schizonts) [111]. Gamonts localize to the enterocyte apical cytoplasm near villus tips in the ileum [125]. Macrogamonts (female) are subspherical cells, measuring 8 × 6 µm, with a central nucleus and numerous mitochondria and they mature directly into macrogametes [126]. In contrast, microgamonts (male) are ovoid to ellipsoidal, 6 µm long and 2 µm wide, and undergo nuclear division to produce up to 30 motile microgametes. These microgametes bud from the surface of the microgamont, each equipped with two flagella (~10 μm long) and a single mitochondrion [125]. The fusion of microgamete and macrogamete produces the immature unsporulated oocyst, which is released into the intestinal lumen (by rupture of epithelial cells) and excreted in feces [127]. Oocysts can be excreted in cat feces 3–21 days post-infection, patent period to be less than 1 week, and peak excretion at the 7th day on average [24].

### 3.2. Life Cycle in the Environment

Cat feces contain diploid oocysts that are formed from the fusion of gametes. The multilayered oocyst wall enables survival for over a year under harsh extremes of environment, including mechanical and chemical stress [128]. Under favorable conditions, diploid oocysts sporulate in the environment via meiosis, producing eight haploid sporozoites as follows:

#### Sporulation

Sporulation of diploid oocysts occurs outside the cat after 24 h, depending on aeration and temperature, as follows: The nucleus of unsporulated oocysts divides twice to form four nuclei; and a second limiting membrane is formed surrounding all four nuclei [129]. The cytoplasmic division results in the formation of two sporoblasts, each with two nuclei [130]. Both sporoblasts (within the oocysts) elongate to be converted to sporocysts. Sporozoite formation begins with the appearance of two dense cytoplasmic plaques (anlagen) at opposite poles of the sporocyst. Each of the two original nuclei undergoes division, producing four nuclei that migrate into the elongating anlagen [131]. Via endodyogeny—a specialized form of internal budding characteristic of apicomplexans—these anlage give rise to two mature sporozoites, totalling four per sporocyst that align peripherally within each sporocyst. In a nutshell, each sporulated oocyst (11 × 13 μm) contains two sporocysts (6 × 8 µm), and each sporocyst contains four sporozoites [115]. Sporulated oocysts are more environmentally resistant (can survive for several months) than the unsporulated oocysts, likely due to the structure of inner layers of the oocysts and sporocysts [113].

### 3.3. Life Cycle in Intermediate Hosts, Including Humans and Cats

Toxoplasmosis spreads in intermediate hosts (including humans and cats) by ingestion of *T. gondii* sporulated oocysts from contaminated food or water, by eating undercooked or raw meat containing tissue cysts from chronically infected ones (humans, other mammals, and birds), by receiving the infected organ/blood from the infected individual, through transplacental transmission, accidental (laboratory) exposure [13,110,113,132], and through milk from the infected mother [133].

Proteolytic enzymes, low gastric pH, and parasite-derived proteases degrade the cyst/oocyst wall, releasing bradyzoites from tissue cysts and sporozoites from oocysts [114].

Felines fed bradyzoite tissue cysts will probably have tachyzoites within a day [24]. Excysted sporozoites invade various nucleated cells [134], except erythrocytes (irrespective of the species having nucleated or anucleated erythocytes) [135], and form a parasitophorous vacuole. The rapidly dividing tachyzoite stage of *T. gondii* replicates through endodyogeny within host cell parasitophorous vacuoles. Two daughter tachyzoites form internally while maintaining the mother cell’s structure, emerging through outer membrane incorporation. This process generates the characteristic rosette clusters observed during acute infection [136]. Tachyzoite replication continues until it reaches a critical mass, which leads to their egress from the vacuole. The host cell is destroyed, and the released tachyzoites infect adjoining cells. Tachyzoites disseminate freely within phagocytic cells or via lymphatics. Tachyzoites can cross epithelial and endothelial barriers. In mice, a sporozoite takes 12 h post-infection to divide into two tachyzoites.

Tachyzoites efficiently invade nearly all nucleated cells of homeotherms, causing acute infection [114,115]. Unlike schizonts in enterocytes, tachyzoites replicate in the lamina propria via endodyogeny (leading to formation of two daughter cells) [124]. This (tachyzoite) is the stage of *T. gondii* that is mainly cultivated and maintained in human foreskin fibroblast (HFF) cells for in vitro research purposes under Biosecurity Level 2 conditions [4].

**Figure 1:**
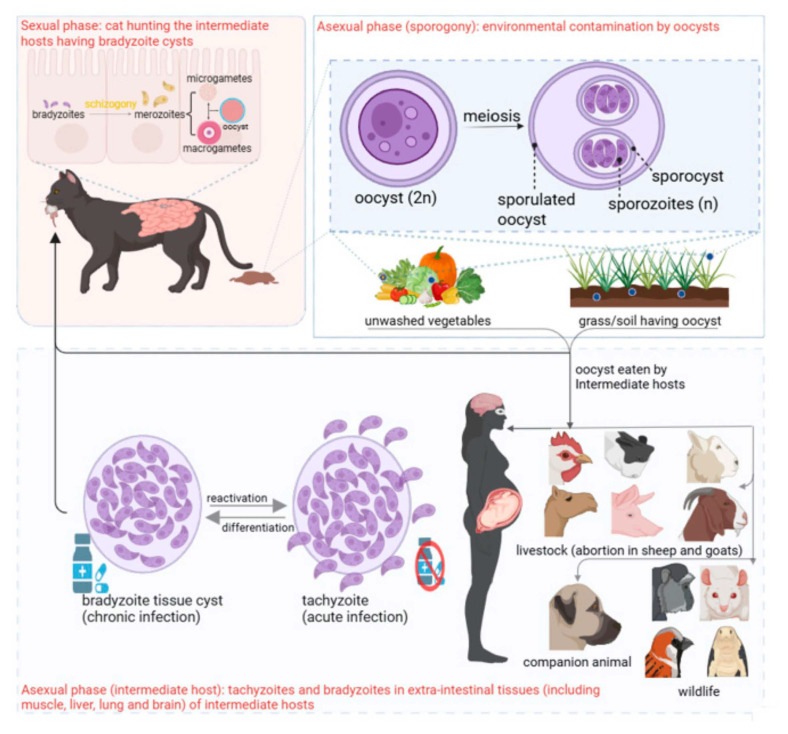
Life cycle of *T. gondii*. Created in BioRender. Farhab, M. (2025) https://BioRender.com/n4n4txj.

Tachyzoites can differentiate into slow-growing bradyzoites within tissue cysts, via endopolygeny, resulting in chronic infection [137]. The bradyzoite tissue cysts vary in size from 5 to 50 µm in diameter [115]. Felines fed tissue cysts will probably have bradyzoite tissue cysts within 7-21 days. They are mostly formed in the brain, but they can also be formed at several other tissues (as the sclera, optic nerve, tongue, etc.) of the intermediate host during chronic infection [12]. Ingesting the tissue cysts leads to the continuation of the sexual cycle and the asexual cycle within the cats, while intermediate hosts support only the asexual cycle [115] as presented in Figure 1.

### 3.4. Pathogenesis in General

The bradyzoites/sporozoites, released from the ingested tissue cysts or oocysts, penetrate and replicate in the intestinal epithelial cells, and are transformed to tachyzoites. A host may die because of necrosis of the intestine and mesenteric lymph nodes before other organs are severely damaged. Tachyzoites then disseminate to a variety of organs, causing fatal interstitial pneumonia in felids [13,110].

In the immunocompetent host, both the humoral and the cellular immune responses are activated to limit the infection as the activation of antibodies, macrophages, interferon γ, and CD8+ cytotoxic T lymphocytes. These antigen-specific lymphocytes are capable of killing both extracellular parasites and target cells infected with parasites [138,139]. Parasite dissemination initiates the production of IL-12, IL-18, and IFN-γ, leading to Th1 activation by the host, and this immune response is sufficient in almost all immunocompetent individuals to cope with this foreign invader [108,140]. The role of specific cytokines against *T. gondii* is summarized in Table 4.

As tachyzoites are cleared from the acutely infected host, tissue cysts containing bradyzoites begin to appear in various body tissues of the host. *Toxoplasma gondii* secretes signaling molecules into infected host cells to modulate host gene expression, metabolism, and immune response [141]. CD8+ T cells and alternatively activated macrophages can kill cysts at least in the murine model, but it requires further validation [142].

Immuno-compromised hosts allow the persistence of tachyzoites and give rise to progressive focal destruction in affected organs [143,144]. All infected individuals harbor cysts containing bradyzoites (but as subclinical infection), bradyzoites within these cysts replicate to a threshold after which the cysts are ruptured, which leads to the liberation of bradyzoites. These bradyzoites then invade other tissues, leading to development of new bradyzoite-containing cysts [145]. This is the probable source of recrudescent infection in immune-compromised individuals and the most likely stimulus for the persistence of antibody titers in the immune-competent host. Although the concept that toxoplasmosis is related to neuropsychiatric conditions is not proven yet [146,147,148,149]. In rodents, the claim that chronic *T. gondii* infection increases predation also needs further confirmation [150,151].

**Table 4:** Role of cytokines against *T. gondii*.

Cytokine	Main Functions	Ref.
INF α	Induce other inflammatory proteins.	[152]
INFγ	Provides protection against *T. gondii* by activation of MΦ, NO, and GTPase signaling. Also induces cell-autonomous immunity, iNOS, and IDO production.	[153]
TNFα	Involved in an acute inflammatory response.	[153]
IL1β	Acute phase response mediator; induces other inflammatory proteins.	[154]
IL2	Induces growth of T cells and the release of IFNγ, involved in the lytic activity of MΦ and NK cells.	[155]
IL4	Antagonizes the products of Th1 cells; long exposure leads to chronic toxoplasmosis.	[156]
IL5	Has a counter-protective role in acute toxoplasmosis and a protective role in chronic toxoplasmosis.	[157]
IL6	Has a pleiotropic role in immunity, including creating barriers in early ocular toxoplasmosis, enhancing activities of NK cells, and maturation of T/B cells.	[158]
IL7	Plays a crucial role in the development of memory CD8+ T cells.	[159]
IL10	Suppress inflammation to prevent *T. gondii* encephalitis, Controls hyper-inflammation, regulates the protective functioning of CD4+ cells, and plays a suppressive microbicidal function for MΦ and Np.	[160]
IL12	Central inducer of IFNγ, activates NK cells, CD4 T cells, and CD8 T cells.	[161]
IL15	Required for optimal role of NK cells, CD8+ cells, and IELs.	[162]
IL17A	Mainly involved in innate immunity by the recruitment of Np IL12, IFNγ, and IL6.	[163]
IL18	Involved in the production of IFNγ by NK cells and T cells.	[164]
IL23	Stimulates NK cells and T cells more specifically in the absence of IL-12.	[165]
IL27	Required for resistance to chronic toxoplasmic encephalitis, induces CXCR3, T-bet, Blimp1, and IL10 expression, inhibit Th17 development.	[166]
IL33	induces CCL 2 expression (proinflammatory), induces IL-10 production by M2 macrophages (anti-inflammatory).	[167]
TGFβ	Anti-inflammatory role in the brain, eyes, and intestine.	[168]

IFN (Interferon), IL (Interleukin), TNF (Tumor Necrosis Factor), TGF (Transforming Growth Factor), MΦ (macrophages), Np (neutrophils), IELs (intraepithelial lymphocytes).

## 4. Epidemiology of Toxoplasmosis

**Figure 2:**
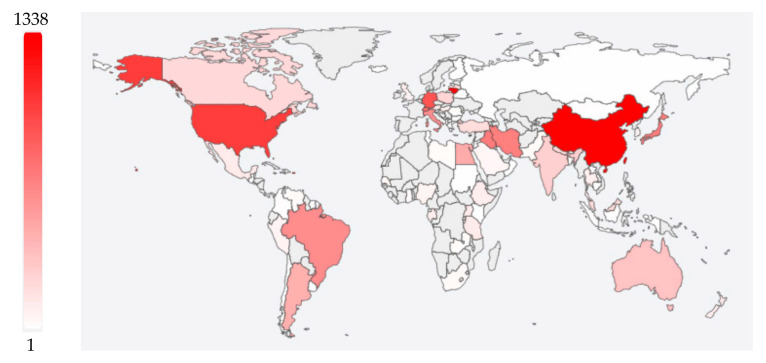
Epidemiology of *T. gondii*. Countries with more PopSet, including humans and animals, are depicted in darker red, while those with fewer reported cases are indicated with a faint red color. Countries shown in the map with the same color as the background indicate that no PopSet data was available from these countries in ToxoDB. Data collected from [8].

**Figure 3:**
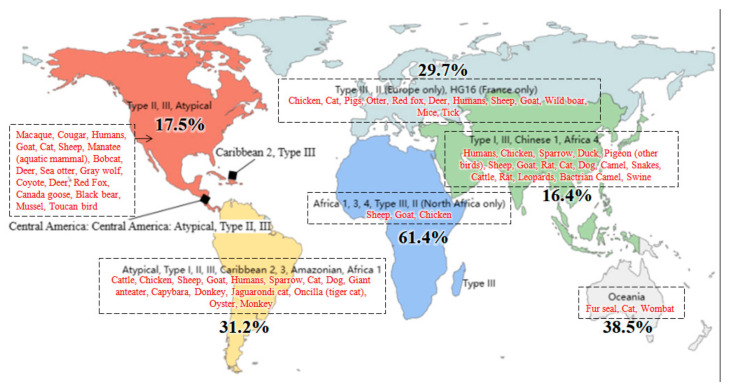
Global distribution of *T. gondii* groups defined by microsatellite typing using 15MS.

Toxoplasmosis has a global distribution. The infection rate is much higher in developing and underdeveloped countries compared to developed countries [169]. The highest PopSet counts are reported from China and the United States to be 323 and 283, respectively, while the lowest (1 each) are from Costa Rica and Egypt, as presented in Figure 2. Continent-wise, prevalence is the highest in Africa (61.4%), intermediate in Oceania (38.5%), South America (31.2%), and Europe (29.7%), and the lowest in North America (17.5%) and Asia (16.4%), as presented in Figure 3. Prevalence is influenced by factors such as climatic conditions and cat populations [123,170]. The survival rate of oocysts is strongly affected by several climatic and environmental factors. Prevalence is higher in older people compared to the young. In addition, socio-economic factors also play a significant role in transmission [123].

*Toxoplasma gondii* is a single species with diverse isolates and strains, categorized into Types I, II, III, and atypical variants based on molecular markers and murine virulence. Type I isolates are considered virulent to outbred mice, whereas Type II and III isolates are not [49]. Although many other types also exist, having comparatively less prevalence or encompassing less geographical region as HG16, Caribbean 2, Chinese 1, Africa 1, and Africa 4. In general, Type I stains are rare, while Type II and Type III have global prevalence (except in Brazil) [5]. The *T. gondii* strains in Europe and North America are similar to each other. Africa 1 is reported in sub-Saharan Africa [5,171,172,173] and in Denmark. HG16 is prevalent only in France, Caribbean 2 is prevalent only in the Caribbean region, Chinese 1 and Africa 4 are prevalent in China [174]. We have accessed the data of *T. gondii* prevalence in different species of different countries through https://toxodb.org (accessed on 6 June 2025) and presented in Figure 3. Notably, high PopSet counts are reported from the United States and China (regions with low prevalence), while Africa (with the highest prevalence) reports nearly the lowest PopSet counts (Figure 2 and Figure 3).

## 5. Clinical Signs

The severity of the disease varies in different species and the infected individual remains a carrier for life. Most host species (including healthy humans) are asymptomatic; on the one hand, it is fatal to marsupials, neotropical primates, and some marine mammals on the other hand [2,94,175,176,177]. Severity depends on the frequency of exposure, inoculum size, life cycle stage, parasite strain, route of infection, host genetics, unrecognized immune deficiencies, geographic location, and variability of immune responses of the affected host [178].

### 5.1. Clinical Signs of Toxoplasmosis in Humans

Although immunocompetent persons are usually asymptomatic or have mild symptoms, presenting with nonspecific symptoms such as fever, headache, fatigue, and lymphadenopathy, their increased susceptibility to neuropsychiatric disorders such as autism, schizophrenia, anxiety, and Alzheimer’s disease is notable [179,180].

The trimester of gestation significantly influences congenital infection outcomes. Infection with *T. gondii* before pregnancy confers little or no risk to the fetus except in women who become infected up to 3 months before conception [181]. Of the children infected with congenital toxoplasmosis, seroconversion and transmission to the fetus occurred in more than half of the cases during the third trimester, followed by less than a fourth in the second trimester and <3.5 percent in the first [182]. First-trimester transmission may cause miscarriage, stillbirth, and/or severe neurologic sequelae [182,183,184]. First trimester shows reduced Toll-like receptor expression in trophoblast cells, indicating a reduced immune response of the placenta to intrauterine infection [185]. So, first-trimester infections are associated with more severe fetal damage such as chorioretinitis, hydrocephalus, and cerebral calcifications. Maternal infection in the third trimester often results in asymptomatic newborns. However, if not treated appropriately, these newborns might develop retinochoroiditis and neurologic deficits in childhood or early adulthood [181,182,185]. If the infected individuals are not treated, then chorioretinitis may develop as a sequel [132]. The parasite persists in the brain for the lifetime of infected individuals [186].

In France, most strains isolated from congenital infections are Type II and the severity of congenital toxoplasmosis is related to the trimester of pregnancy when the mother becomes infected. In Brazil, third-trimester maternal infection often leads to severe congenital toxoplasmosis, linked to atypical genotypes [185].

In pregnant women, the placenta is a target tissue for parasite multiplication, and aids in trans-placental transmission [7]. So, as pregnancy progresses, the placenta development increases, and hence the infection rate is low at the earlier stages of pregnancy and high at later stages of pregnancy [123,187,188]. But the outcome of the infection during early gestation is more severe, and vice versa. Without treatment, the rate of fetal transmission during the first, second, and third trimesters averages up to 25%, 50%, and 70% [123]. Treatment of the pregnant woman reduces the incidence and severity of manifestations of congenital infection [189].

Ocular toxoplasmosis causes 35% of chorioretinitis cases in the United States and Europe [190]. Both congenital transmission and postnatal exposure contribute to onset of ocular toxoplasmosis. An infected individual may present clinical signs such as eye pain, scotomas, blurred vision, photophobia, and with macular involvement threatening central vision, extraocular muscle involvement leading to strabismus, and yellow-white cotton-like patches in the posterior retina [191]. Congenital cases show chorioretinal scarring with fibrosis. Immunocompromised individuals develop severe necrotizing retinitis with both tachyzoites and bradyzoite cysts. Eye infections from toxoplasmosis can be an early warning sign that the parasite may soon attack the brain, causing life-threatening encephalitis, warranting neurologic evaluation. Although initial inflammation may resolve, recurrent chorioretinitis frequently causes cumulative retinal damage and secondary glaucoma [192,193].

Clinical signs such as lymphadenopathy may be self-limited or prolonged symptoms. The signs are usually resolved within a few months to more than a year [194]. The lymph nodes mostly involved are the cervical nodes (present as non-tender, discrete, firm, not matted or fixed to contiguous tissues and do not suppurate), mesenteric nodes (pain, fever may be mistaken for appendicitis), and pectoral nodes (may be mistaken for breast cancer) [195,196].

More rarely, seropositive transplant recipients may reactivate their latent infection due to the transplant-related immune suppression, although typically, if a seropositive person receives a transplant from a seropositive donor, there is a rise in IgG antibody titer and development of IgM antibody specific for *T. gondii* without illness ascribed to the parasite [195]. Toxoplasmosis is a rare but frequently fatal complication in bone marrow transplant recipients, particularly when the donor is seronegative for toxoplasmosis. The lack of donor-derived immunity can result in an 80% mortality rate. Early diagnosis remains challenging, with only 47% of cases identified ante-mortem, and pulmonary or cerebral infections are associated with the poorest outcomes. Given these risks, prophylaxis is strongly recommended for high-risk serodiscordant pairs (donor-negative/recipient-positive) to prevent lethal infection [197].

Individuals with AIDS who are seropositive for *T. gondii* are at high risk for *T. gondii* encephalitis (TE), mostly due to recrudescent infection [198] and when the CD4+ T-cell count falls below 100/μL. Other clinical findings include meningoencephalitis (meningeal involvement is uncommon), altered mental status (75%), seizures (33%), motor deficits, cranial nerve palsies, movement disorders, dysmetria, visual-field loss, aphasia, ataxia, hydrocephalus, choreiform movements, choreoathetosis, and necrotizing encephalitis caused by direct invasion by the parasite [198]. Clinical signs of human toxoplasmosis are summarized in Table 5.

**Table 5:** Clinical manifestations within humans.

System Involved	Pregnant Women [7]	Fetus/Infant [199]	Older Children and Adults (Immunocompetent) [200]	Immunocompromised (HIV) [201]	Cardiac/Renal Transplant [202]	Bone Marrow/HSCT Transplantation [203]
Neurological	Same as in older children and adults	Encephalitis, epilepsy, psychomotor retardation, microcephaly, cerebral calcification, hydrocephalus	Encephalitis, meningoencephalitis, meningitis, fatal brain abscess, seizures, poor cognition/motor function	Encephalitis, hemiparesis, altered mental state, seizures, cranial nerve disturbances	Encephalitis (within 3 months post-transplant)	Localized encephalitis, seizures, headache, confusion
Ophthalmologic	Retinitis, chorioretinitis, peripheral retinal scars, uveitis	Retinochoroiditis, vitritis, blurred vision, scotoma, photophobia, strabismus, glaucoma, vision loss	Retinitis, retinochoroiditis, branch retinal artery occlusion, risk of permanent vision loss	Ocular involvement	Retinal involvement	-
Cardiovascular	Same as in older children and adults	Myocarditis, pericarditis	Myocarditis, pericarditis	-	Myocardial involvement	-
Respiratory		Pneumonia	Diffuse interstitial pneumonia	Febrile illness with cough, dyspnea	Pulmonary involvement	
Musculoskeletal		Myositis, dermatomyositis	Polymyositis, dermatomyositis, myositis, myalgias	-	-	-
Gastrointestinal (GI)/Hepatic		Jaundice	Pancreatitis, increased liver enzymes, mesenteric lymphadenopathy, GI pathologies, hepatocellular abnormalities	-	-	-
Hematologic/Systemic	Asymptomatic to illness, cervical lymphadenopathy	Rash, petechiae, anemia, high mortality risk	Sepsis-like syndrome, weight loss	-	Fever	Fever
Other	-	-	Guillain–Barre syndrome	Psychosis, dementia, anxiety	-	-
Timing/Special Notes	Can show all signs of other groups	Progressive manifestations	-	Brain predominantly infected	Reactivation infection (3 months post)	Often leads to death

HSCT: hematopoietic stem cell transplantation; GI: gastrointestinal.

### 5.2. Clinical Signs of Toxoplasmosis in Animals

Table 6 and Table 7 summarize species-specific clinical manifestations of toxoplasmosis in animals, excluding nonsystemic signs (e.g., fever, anorexia).

#### 5.2.1. Clinical Manifestations Within Wildlife

Wildlife susceptibility to *T. gondii* ranges from resistant carriers to highly vulnerable species. Resistant hosts rarely show symptoms but may shed oocysts (felids) [150] or maintain latent infections (capybaras) [204]. Endangered species such as striped skunks [205], capybaras, and nutria act as resistant reservoir hosts. While most bat species show no disease, juvenile flying-foxes develop acute pneumonia [206]. Rodents range from fatally affected woodchucks to completely resistant nutria [204,207]. Moderately susceptible species (deer) survive with sporadic acute disease [208], while highly susceptible groups (lagomorphs [209], birds of prey [210]) suffer organ-specific necrosis. Extremely vulnerable animals (koalas [211] and otters [212]) often die rapidly from systemic collapse. New World primates show higher susceptibility than Old World primates, rarely surviving the disease [213] (Table 6).

**Table 6:** Clinical manifestations within wildlife.

Class	Order Family	Species and Clinical Manifestations	Ref.
**Mammalia**	Carnivora Felidae	Wild cats (*Felis silvestris*): acute infection. Captive pallas’s cats (*Otocolobus manul*): up to 60% of infected kittens die, repeated transplacental transmission. Sand cats (*Felis margarita*). Gordon’s wildcat (*Felis silvestris gordoni*).	[128,214]
	Mephitidae (skunks)	Striped skunk (*Mephitis mephitis*): asymptomatic.	[205]
	Ursidae	Giant panda (*Ailuropoda melanoleuca*): affects gastrointestinal, and respiratory systems which can lead to death.	[64]
	Mustelidae (sea otters)	Southern sea otters (*Enhydra lutris nereis*): significant mortality with encephalitis.	[212]
	Rodentia Sciuridae (squirrels)	Red squirrels: fatal acute toxoplasmosis, mimic signs of rabies. Eastern fox squirrel (*Sciurus niger*): pneumonitis. Eurasian red squirrel (*Sciurus vulgaris*): necrosis in the spleen, liver, and lungs. Swinhoe’s striped squirrel (*Tamiops swinhoei*): enteritis.	[68]
	Castoridae (beavers)	Beaver (*Castor canadensis*): fatal systemic toxoplasmosis (lymphohistiocytic encephalitis, myocarditis, interstitial pneumonia with multinucleated cells).	[215]
	Sciuridae	Woodchuck (*Marmota monax*): head tilt, circling, and rapid weight loss.	[204,207]
	Caviidae	Capybara (*Hydrochoerus hydrochaeris*): no clinical toxoplasmosis.	
	Echimyidae	Nutria (*Myocastor coypus*): no clinical toxoplasmosis.	
	Lagomorpha Leporidae (rabbits, hares)	Domestic rabbits (*Oryctolagus cuniculus*): die with or without present diarrhea, foci of necrosis of the spleen and liver associated with the massive presence of multiplying tachyzoites. Brown hares (*Lepus europaeus*): develop fatal, hemorrhagic enteritis, enlargement and hyperemia of mesenteric lymph nodes, splenomegaly, and multiple necrotic lesions in the parenchyma of the liver and other organs, and death. Mountain hare (*Lepus timidus*): gross lesions and extensive necrotic areas in the small intestine, mesenteric lymph nodes, and liver.	[216]
	Eulipotyphla Talpidae (moles/Insectivores)	Mole (*Talpa europaea*): fatal toxoplasmosis, affecting the brain. White-toothed shrews (*Crocidura russula*): affect brains and hearts. Striped field mice (*Apodemus agrarius*): affect heart.	[217]
	Chiroptera Pteropodidae (bats)	Spectacled flying-fox (*Pteropus conspicillatus*): acute toxoplasmosis Little red flying-fox (*Pteropus scapulatus*): acute toxoplasmosis with severe, acute interstitial pneumonia and fibrin present within alveoli in the lungs, multiple foci of gliosis, including gemistocytic astrocytes, at all levels of the cerebrum, cerebellum, and brainstem. Red night bats: no clinical disease.	[206]
	Artiodactyla Cervidae (deer)	No transplacental infection unless an acute infection occurs during pregnancy. Acute toxoplasmosis and death in mule deer. Reindeer (*Rangifer tarandus*): enteritis and death.	[218]
	Bovidae (Antelope, nilgai)	Pronghorn antelope (*Antilocapra americana*): acute fatal toxoplasmosis. Rocky Mountain bighorn sheep (*Ovis canadensis canadensis*): encephalitis. captive nilgais (*Boselaphus tragocamelus*): abortion and neonatal death.	[51,214]
	Non-human primates Callitrichidae	Golden lion tamarins (*Leontopithecus rosalia*): fatal.	[214,219]
	Cercopithecidae (Macaques)	Rhesus monkey (*Macaca mulatta*): congenital toxoplasmosis. Stump-tailed macaques (*Macaca arctoides*): congenital toxoplasmosis. Cynomolgus monkey (*Macaca fascicularis*): recurrent toxoplasmic retinochoroiditis. Barbary macaque (*Macaca sylvana*): concurrent central nervous system toxoplasmosis.	
	Atelidae	* Alouatta* sp. Diffuse yellowish liver with multifocal petechiae, enlarged spleens with bulging edges and ared pulp expansion, pulmonary edema, intestinal mucosal petechiae and suffusions.	[100]
		○ *Alouatta belzebul*: prostration, diarrhea.	[60]
		○ *Alouatta caraya*: prostration, inappetence, abdominal distension and pain, intestinal hypomotility.	[220,221]
		*Brachyteles arachnoides*: prostrated, laterally recumbent, tachypnea, pyrexia, bilateral pneumonia, cardiovascular collapse, and died.	[222]
		*Alouatta guariba guariba*: mild jaundice, depression, anorexia, fever, oliguria, and severe hepatosplenomegaly, hepatomegaly with yellowish discoloration.	[223]
		*Lagothrix lagothricha*: apathetic, diarrhoea, dark coloured urine, vomit, death.	[98]
	Cebidae	Cebus capucinus: paraparesis, interstitial pneumonia, hepaticnecrosis, encephalitis, and enteritis.	[224]
		Squirrel monkey (*Saimiri sciureus*): acute fatal. Inflamed liver and spleen, enteritis, villus atrophy, multifocal areas of necrosis along with *T. gondii* in the lungs, liver, spleen, lymph nodes, adrenal glands, heart, congested and pulmonary edema, respiratory failure.	[5,38,225,226]
	Diprotodontia Macropodidae	Acute fatal, respiratory distress, diarrhoea, neurological disturbances, myocardial hemorrhages and pale streaks, lymphadenomegaly, splenomegaly, adrenal enlargement and reddening, gastrointestinal reddening and ulceration, pancreatic swelling, brain malacia. Black-faced kangaroos (*Macropus fuliginosus melanops*): Congenital toxoplasmosis. Tammar wallabies (*Macropus eugenii*): acute infection, necrosis, enteritis, lymphadenitis, adrenalitis, carditis, myositis, and encephalitis. Severe generalized pulmonary congestion and edema.	[227,228,229,230,231,232]
	Phascolarctidae	Koalas (*Phascolarctos cinereus*): acute fatal, myocardial hemorrhages, and pale streaks.	[233,234]
	Vombatidae	Wombats (*Vombatus ursinus*): respiratory distress, neurological disturbances.	[234]
	Phalangeridae (Possums)	Acute fatal, respiratory distress, neurological disturbances, myocardial hemorrhages and pale streaks, splenomegaly, gastrointestinal reddening and ulceration, brain malacia.	[234,235,236]
	Peramelemorphia Peramelidae (Bandicoots)	Neurological disturbances, adrenal enlargement and reddening, pancreatic swelling. Eastern barred bandicoots (*Perameles gunnii*): acute infection, necrosis, enteritis, lymphadenitis, adrenalitis, carditis, myositis, and encephalitis. Severe generalized pulmonary congestion and edema.	[237,238,239]
	Dasyuromorphia Dasyuridae Myrmecobiidae	Acute fatal, neurological disturbances, gastrointestinal reddening, and ulceration	[5]
	Peramelemorphia Thylacomyidae	Bilby (*Macrotis lagotis*): neurological disturbances, adrenal enlargement and reddening, pancreatic swelling.	[240]
	Cetacea Delphinidae	Bottlenose dolphin (*Tursiops aduncus*): congenital toxoplasmosis. Risso’s dolphin (*Grampus griseus*): disseminated congenital toxoplasmosis.	[68]
**Aves**		Hawaiian crow (*Corvus hawaiiensis*): fatal. Black-footed penguin (*Spheniscus demersus*): fatal, nervous signs. Bald eagle (*Haliaeetus leucocephalus*): necrotizing myocarditis. Barred owl (*Strix varia*): hepatitis. Turkeys: fatal systemic toxoplasmosis.	[214,241]

#### 5.2.2. Clinical Manifestations Within Domestic Animals

The cats may remain asymptomatic or develop systemic illness, with rare cases of transplacental/lactogenic transmission [24,177,242]. Dogs demonstrate mild respiratory or hepatic involvement and no evidence of vertical transmission [243]. Ferrets can have congenital infection. Ectothermic pets do not experience natural *T. gondii* infections, despite being experimentally infectible in laboratory settings [69]. *Toxoplasma gondii*’s agricultural impact concentrates primarily in small ruminants [244] and swine [245], while most other farm species either resist infection or serve as silent reservoirs, except camels, which may develop acute respiratory disease [246]. Poultry species show decreased egg production, and turkeys harboring tissue cysts in their muscles without clinical signs [12,247,248] (Table 7).

**Table 7:** Clinical manifestations within domestic animals.

Domestic Animals		
Pet animals		
Cats (definitive host)	Asymptomatic or polypnea, icterus, uveitis and retinochoroiditis, pericardial and abdominal effusions, diffuse necrotizing hepatitis, transplacental and lactogenically.	[24,177,242]
Dogs	Respiratory and hepatic systems, no transplacental infection, resistant to experimental toxoplasmosis.	[243]
Ferrets	Congenital toxoplasmosis, acute and chronic forms.	[69]
Mink	Can be naturally infected.	[71]
Fish, reptiles, amphibians	Not occur in fish, reptiles, or amphibians as natural infection, but they can be experimentally infected.	[4,249]
Production Animals		
Horses	Relatively resistant to experimental infection, no clinical disease.	[17,18]
Swine	Can be naturally infected, causing sow abortion (sows abort only once).	[245]
Cattle	Rare, no report of zoonotic.	[16]
Sheep, goats	Abortion.	[244]
Buffalos	No clinical disease.	[250]
Camels	Acute toxoplasmosis with dyspnea.	[246]
Llamas, alpaca, and vicunas	No clinical disease.	[251]
Chickens	No clinical signs, no vertical transmission, decreased egg production.	[247]
Turkeys	No clinical signs, having tissue cysts in breast and leg muscles.	[248]
Ducks and geese	No clinical disease.	[12]

Clinical signs of serologically positive species, not supported with confirmatory tests of toxoplasmosis, are not listed in this table.

## 6. Diagnosis of Toxoplasmosis

### 6.1. Diagnosis of Toxoplasmosis in Animals

Toxoplasmosis is diagnosed by biologic, serologic, or histologic and molecular methods, or some combination of these [252]. Diagnosis in felids can also be performed through coproparasitological examinations [253]. While traditional serological tests such as the indirect hemagglutination assay (IHA), indirect fluorescent antibody test (IFAT), and latex agglutination test (LAT) have been historically used for toxoplasmosis diagnosis, many of these are now considered outdated. Serological testing remains a cornerstone for *T. gondii* diagnosis in veterinary medicine, serving both clinical and epidemiological applications. While newer molecular methods have emerged, well-established serological assays continue to provide reliable diagnostic solutions. The indirect fluorescent antibody test (IFAT) maintains particular utility due to its ability to differentiate acute and chronic infections through IgM and IgG detection, with reported sensitivity of 85-95% in domestic species. Similarly, the modified agglutination test (MAT) is widely employed in veterinary laboratories, offering excellent specificity (92-98%) and stability of reagents for field surveys. These techniques complement newer diagnostic approaches while remaining indispensable for (1) large-scale seroprevalence studies, (2) routine screening in clinical practice, and (3) situations where tissue sampling is impractical. Their continued use reflects standardized protocols, cost-effectiveness, and proven performance across animal species. Currently, enzyme-linked immunosorbent assay (ELISA) remains widely used due to its reliability and automation potential [173]. However, newer advanced techniques—including chromatographic immunoassays (e.g., lateral flow tests), chemiluminescence assays (CLIAs), and real-time PCR—have improved sensitivity and specificity for detecting *T. gondii* infection, particularly in congenital and immunocompromised cases [252]. Increased IgM titers (>1:256) are consistent with recent infection. IgG titers must be measured in paired serum samples obtained during the acute and convalescent stages (3–4 weeks apart) and must show at least a fourfold increase/decrease in titer [254,255] (Table 8 and Table 9).

Histological examination may present tachyzoites or bradyzoites in domestic animals, but it is not definitive due to the morphological overlap with other cyst-forming coccidia (*Neospora caninum*, *Hammondia* spp.). Thus, histopathology must be combined with *T. gondii*-specific immunohistochemistry (targeting *SAG1*/*BAG1* antigens) and PCR amplification of conserved genomic targets (*B1/REP-529*), particularly in food animals where accurate speciation carries zoonotic significance.

Application of specific polymerase chain reaction (PCR) assays allows diagnosis from tissue DNA samples [256]. Isolation of *T. gondii* from secretions, excretions, and organs by bioassay using laboratory animals is an accurate method of diagnosing animal infection. It has the disadvantage that it is difficult to perform, less sensitive, and unless the *T. gondii* strain is highly virulent, it requires three weeks before mouse examination yields recognizable *T. gondii* cysts [257].

### 6.2. Diagnosis of Toxoplasmosis in Humans

The diagnosis of parasite infections is typically divided into direct methods—such as microscopy, molecular and imaging techniques, and biological isolation—and indirect methods, including serological tests like Sabin–Feldman dye test, IFA, ELISA, and agglutination tests for antibody detection [258].

Microscopy detects tachyzoites or tissue cysts. Tachyzoites are more likely to be detected in acute infection, but they are often cleared before sampling. Conversely, bradyzoite tissue cysts are sparse and unevenly distributed, making them difficult to identify even with the use of specialized stains [198]. The main techniques include the following: (1) Giemsa or H&E staining for tachyzoites and cysts, (2) PAS staining for bradyzoites, and (3) immunohistochemistry (IHC) with *T. gondii*-specific antibodies (anti-*SAG1* for tachyzoites, anti-*BAG1* for bradyzoites) [258,259]. This morphological approach is particularly useful for tissue diagnosis in immunocompromised patients but remains less sensitive (30-50%) than PCR or serology [198] due to (a) sampling limitations of focal infections, (b) intermittent parasite presence, and (c) technical challenges in distinguishing *T. gondii* from similar structures. In general, microscopic detection of *T. gondii* cystic forms in biological samples from patients with suspected acute toxoplasmosis is difficult. Conversely, rapidly proliferating forms are more likely to be detected.

The Sabin–Feldman dye test, historically vital for toxoplasmosis diagnosis, detects serum antibodies with high sensitivity across species. However, its reliance on live *T. gondii* tachyzoites posed biosafety risks and technical challenges, leading to its obsolescence [30]. Subsequent serological advances included the indirect immunofluorescent antibody test (IFA), which eliminated the need for live parasites and became a preferred alternative [260].

Modern diagnostics now prioritize enzyme-linked immunosorbent assay (ELISA) based techniques. Unlike older methods, ELISA platforms use purified or recombinant antigens (e.g., *SAG1*, GRA7) to detect antibodies, enhancing safety, reproducibility, and scalability. Among ELISA variants, IgG ELISA is now the most widely used for chronic infection screening, owing to its ability to quantify long-term antibody responses with high specificity [261]. For acute infection confirmation, testing paired sera (collected 1–2 weeks apart) to detect rising IgG titers remains critical, supplemented by IgM ELISA (to identify recent exposure) and IgG avidity testing (to distinguish acute from past infections). Further advancements include chemiluminescence assays (e.g., Architect, Liaison), which offer automated, high-throughput analysis with improved sensitivity and standardization [257,262].

**Table 8:** Diagnostic potential of immunoglobulins.

Antibody	Life Span	Prediction	Ref.
IgE	Short-term (days to weeks)	Acute toxoplasmosis	[263]
IgA	Few weeks	Supports acute/reactivated/congenital infection	[264]
IgM	1 week to months/years	Congenital toxoplasmosis; alone insufficient to establish acute toxoplasmosis	[265,266]
IgG	Lifelong (post-infection)	Seroconversion and exposure (timing unclear without avidity testing)	[267]

**Table 9:** Summary of direct and indirect, serological, and salivary methods for detection of *T. gondii* infection.

^1^ Type	Method Category	Specific Techniques		Target	Detected Analyte (Key Reagent)
**Direct**	**Microscopy (MS)**	Light MS (Giemsa, H&E, PAS)		Tachyzoites, tissue cysts	Tachyzoites, tissue cysts
		Immunohistochemistry (IHC)		*T. gondii* antigens	Fluorophore-labeled anti-*T. gondii* antibodies
		Electron microscopy		Ultrastructural parasite features	N/A (morphology only)
	**Bioassay**	Mouse bioassay		Viable parasites	N/A (relies viable infection)
		Cell culture		Replicating tachyoites	N/A
	**Imaging**	CT/MRI (CNS lesions)		Brain abscesses, calcifications	N/A
		Ultrasonography (congenital)		Fetal abnormalities	N/A
	**Molecular**	PCR, qPCR, LAMP, etc.		DNA regions	DNA Region
	**Nanoparticle**	Piezoelectric immunoagglutination (PIA)		Antigens	IgG
		^1^ Plasmonic gold chips (PGC)		Antigens (saliva)	IgG
		Quantum dot-labeled antigen detection		Antigens	
	**Immunoassays**	Immunofluorescence antigen detection (IFA-D)		Antigens in tissues	Fluorophore-labeled anti-*T. gondii* antibodies
**Indirect**	**Serological Assays (Antibody Detection)**	Dye test (DT)		Live tachyzoite	IgG, IgA, IgM
		Modified agglutination test (MAT)		Formalin-fixed tachyzoite	IgG
		Indirect fluorescent antibody test (IFAT)		Fixed tachyzoites	IgG, IgM
		Indirect hemagglutination (IHA)		Tanned red blood cells sensitized with soluble antigens	IgG
		ELISA	- Whole-cell lysate	Tachyzoite lysate antigens (TLAs)	IgG
			- Recombinant antigen	*SAG1*/*GRA7*/ROP1 protein	IgG, IgM
			- Multiplex	Multiple antigens	IgG, IgM, IgA
			- Avidity-modified	TLA/recombinant antigens	IgG (avidity index)
			- Chemiluminescence (CLIA)	Recombinant antigens	IgG, IgM
			- Point-of-care	Lateral flow strips	IgG, IgM
		Immunosorbent agglutination assay (ISAGA)		Anti-human IgM	IgM
		Latex agglutination test (LAT)		antigen-coated latex particles	IgG, IgM
		Western blotting (WB)		Tachyzoite lysate/recombinant	IgG, IgM
		Immunochromatographic test (ICT)		^2^ Conjugate or reagent pad	IgG, ^3^ ESA
		Avidity test		Tachyzoite lysate antigen, recombinant antigens	IgG (avidity index)
	**Antigen Detection**	Lateral flow assay			

^1^ All methods are serological except PGC, which uses saliva. PGC is not yet validated for animal diagnosis; ^2^ A conjugate or reagent pad contains antibodies specific to the target analyte conjugated to colored particles (i.e., colloidal gold particles or latex microspheres); ^3^ ESA: excreted/secreted antigens.

A summary of the molecular techniques is presented in Table 10.

## 7. Treatment

### 7.1. Treatment of Toxoplasmosis in Animals

Currently available anti-*T. gondii* drugs limit the multiplication of the tachyzoites and oocyst shedding, but they cannot eradicate the infection [275]. Anti-coccidial drugs treat acute infection. Other drugs, such as diaminodiphenylsulfone, atovaquone, spiramycin, toltrazuril, ponazuril, and diclazuril may also limit infection [5,276,277]. A summary of drugs used to treat animal toxoplasmosis is presented in Table 11.

### 7.2. Treatment of Toxoplasmosis in Humans

In 1953, the traditional gold standard for treating human toxoplasmosis was established—combination therapy targeting two enzymes in the folate pathway: dihydrofolate reductase (pyrimethamine; loading dose of 75 mg followed by 25 mg orally per day) and dihydropteroate synthetase (sulfadiazine; 1 g orally every 6 h), commonly called Pyr-Sulf [31]. Their combination is eightfold more effective than either compound alone [282,283]. However, recent studies highlight alternative regimens (e.g., trimethoprim-sulfamethoxazole, clindamycin-based combinations [284], or atovaquone [282]) with comparable efficacy, particularly in sulfa-intolerant patients or ocular toxoplasmosis. These drugs limit the proliferation of tachyzoites and the destruction of host cells. If fetal infection is detected, the combination of pyrimethamine and sulfadiazine is administered to the mother (only after the first 12–14 weeks of pregnancy) to prevent the severity of congenital toxoplasmosis and to the newborn in the postnatal period [283]. Complete blood counts are monitored twice weekly during Pyr-Sulf treatment [285].

In 1958, spiramycin was found to have antitoxoplasmic activity. It has been used prophylactically in pregnant women as it does not cross the placenta. It is generally effective in reducing fetal transmission of toxoplasmosis when administered early following maternal infection [286], whereas delayed initiation is associated with reduced efficacy and potential adverse outcomes, including fetal loss [184].

Long-term treatment of trimethoprim/sulfamethoxazole can prevent recurrent eye infections; nevertheless, approximately 40% of immunocompromised patients discontinue the medication due to sulfa drug toxicity [287]. Consequently, an alternate treatment that has demonstrated good outcomes is the intravitreal injection of dexamethasone and either clindamycin or trimethoprim/sulfamethoxazole [284].

Pyrimethamine may induce folate deficiency, agranulocytosis, Stevens–Johnson syndrome, toxic epidermal necrolysis, and hepatic necrosis [285]. Leucovorin (folinic acid) (10 mg orally each day for at least 3–6 weeks) may also be added with pyrimethamine and sulfadiazine [288,289,290]. It does not inhibit the action of pyrimethamine on *T. gondii*, as the parasite cannot take up folinic acid at the concentrations achieved in serum. The parasite can take up folate (folic acid), which bypasses dihydrofolate reductase (DHFR) inhibitors or other upstream enzymes [289].

Patients should consume at least 2 L of fluid daily to prevent crystalluria because of sulfadiazine. Fluid intake should be sufficient to ensure a daily urine volume of at least 1.2 L (in adults). Urinary alkalinization may be helpful if urine volume or pH is unusually low [288]. Atovaquone has potent activity against *T. gondii* in patients with trimethoprim–sulfamethoxazole intolerence, and against cyst forms of *T. gondii*, and is effective against *T. gondii* chorioretinitis [282].

In 1973, clindamycin was documented as an alternative for sulfonamide-sensitive patients [34], without loss of efficacy [34,289]. Intravitreal injection of clindamycin and dexamethasone is also being used to treat ocular toxoplasmosis with satisfactory results [284]. It is initially coccidiostatic but becomes coccidiocidal after a few days of treatment. The dosages used against *T. gondii* are higher than for the treatment of anaerobic infections, for which it is marketed [115].

**Figure 4:**
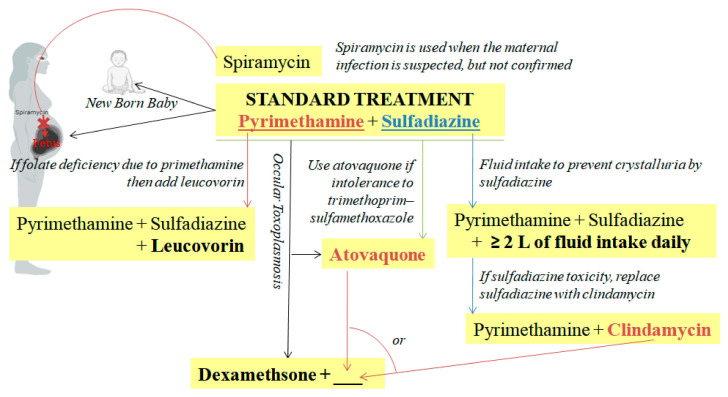
A flow chart of drugs used to treat human toxoplasmosis.

A combination of azithromycin and clarithromycin is also good for treating toxoplasmosis [289]. Virginiamycin, terbinafine, and triazine derivatives (e.g., diclazuril, toltrazuril, ponazuril) can work against toxoplasmosis [291]. A flow chart of drugs used to treat human toxoplasmosis is presented in Figure 4.

There are many limitations of anti-*T. gondii* chemotherapy, including recurrence of the disease, and the solution to this problem is the identification of the population that is at more risk of recurrence, and the application of secondary prophylaxis at the time at which the recurrence is most likely to occur. Also, no drug is available to act against the latent stage of the infection. The treatment success rate remains low, as the drugs available to treat *T. gondii* are active against tachyzoites, and not against bradyzoites [284,292]. Therefore, vaccination against toxoplasmosis could be an effective and appropriate medical prevention [293].

## 8. Control of Toxoplasmosis

### 8.1. Vaccines

As discussed earlier, there are limitations of anti-*T. gondii* chemotherapy, as it may cause the recurrence of the disease, is ineffective against the latent stage of the infection [284], low success rate, may lead to folate deficiency, agranulocytosis, Stevens–Johnson syndrome, toxic epidermal necrolysis, and hepatic necrosis. Therefore, vaccination against toxoplasmosis could be an effective and appropriate preventive measure, especially for specific populations such as pregnant women and HIV patients [284,293].

Despite encouraging results of a live attenuated vaccine (Toxovax^®^, MSD, New Zealand) developed for sheep to limit their abortion storm [294] and recent advances in genetics [295,296,297], no anti-*T. gondii* vaccine is available for humans [298]. Challenges in *T. gondii* vaccine development in humans include, but are not limited to, the complex life cycle of *T. gondii*, diversity of *T. gondii* strains, the establishment of latent infection, and *T. gondii* immune evasion strategies, the difficulty in clinical translation, and lack of optimal adjuvants [293,299]. A summary of proposed different types of vaccines used against toxoplasmosis is presented in Table 12.

### 8.2. Non-Vaccine Prevention Strategies

#### 8.2.1. Food Safety Measures

Methods to control toxoplasmosis apart from vaccination and chemotherapy include proper cooking (≥73 °C, followed by 3 min rest before eating) and freezing of meat (−12 °C for ≥3 days), which kills tissue cysts in contaminated meat [12,115,304]. Freezing meat overnight in a household freezer before human or animal consumption remains the easiest and most economical method of reducing transmission of *T. gondii* through meat. Studies constructed thermal curves showing temperatures required to kill *T. gondii* in infected meat by freezing, cooking, and by gamma irradiation [12].

#### 8.2.2. Personal and Pet Hygiene Practices

With proper hygiene, general interaction with cats (e.g., petting) poses minimal risk. Additional precautions include wearing gloves while gardening, washing hands after handling cats or their belongings, and keeping cats indoors to prevent hunting. Litter boxes should be cleaned daily and pregnant women should avoid cleaning cat litter boxes. Protocols have also been developed for serological screening of pregnant mothers and neonates, but the cost and benefit are poorly documented.

#### 8.2.3. Livestock Management Protocols

On livestock farms, excluding cats is essential to prevent oocyst contamination of feed and water, thereby reducing meat-borne transmission to humans, a significant route of exposure [4]. Effective sanitary planning in animal production is essential to minimize *T. gondii* infection in livestock, which serves as a key source of human transmission [305]. Farms should implement strict biosecurity measures, including rodent control, proper feed storage (to prevent contamination with cat feces), and restricted access to livestock areas to reduce exposure to infected materials. Pregnant livestock (especially sheep and goats) are highly susceptible, with toxoplasmosis causing abortions and economic losses. Vaccination of sheep (where available) and regular serological monitoring can help identify and manage infected herds [306]. Additionally, farm workers should be educated on hygiene practices, such as handwashing and the use of protective equipment, to prevent zoonotic transmission [307]. Proper disposal of placental and fetal tissues from aborted fetuses is crucial to avoid environmental contamination with *T. gondii* oocysts [308].

#### 8.2.4. Feline Management Strategies

Controlling the population of stray cats is a critical measure in reducing environmental contamination with *T. gondii* oocysts, as cats are the definitive hosts of the parasite. Responsible pet ownership programs should emphasize spaying and neutering to prevent overpopulation, alongside public education on proper cat care, including keeping cats indoors to limit their hunting behavior and access to intermediate hosts [309]. Municipalities should support trap-neuter-return (TNR) programs to stabilize feral cat colonies and reduce their numbers [310]. Pet owners should also be encouraged to dispose of cat litter safely—preferably by sealing it in bags before disposal—to prevent oocyst dissemination [311]. Community awareness campaigns can highlight the risks of feeding stray cats near farms or water sources, which may contribute to parasite spread [309].

#### 8.2.5. One Health Implementation

A coordinated One Health strategy, integrating human, animal, and environmental health sectors, is vital for effective toxoplasmosis control. Public health professionals should collaborate with veterinarians on monitoring *T. gondii* prevalence in both pets and livestock, and with environmental agencies to assess contamination risks in soil and water [312]. Educational initiatives can promote safe food handling (e.g., cooking meat thoroughly, washing produce) and protective measures for pregnant women and immunocompromised individuals. Urban planners and policymakers should implement sanitation improvements, such as proper waste management and water treatment, to reduce environmental oocyst persistence. Joint research efforts can identify high-risk transmission pathways and guide targeted interventions. Cross-sectoral cooperation under One Health ensures strategies for mitigating risks at individual and community levels [313,314].

#### 8.2.6. Environmental Conservation

*Toxoplasma gondii* exhibits remarkable genetic diversity in the forest due to high biodiversity, which supports extensive parasite recombination, with wild felids—having larger home ranges than domestic cats—contributing to widespread oocyst contamination through their feces. Importantly, deforestation displaces wild felids into peri-domestic areas, significantly increasing environmental contamination risks near human settlements. Prevention of habitat destruction is therefore crucial to maintain ecological barriers and reduce zoonotic transmission. Concurrently, anthropization favors selection of a few domestic-adapted strains, while forest edges create zones where wild and domestic cycles intersect, facilitating strain mixing [315,316,317].

## 9. Conclusions

The reviewed findings suggest future research should focus on understanding the parasite’s adaptation to diverse hosts, developing screening technologies, and validating animal models. In addition, controlled clinical trials should be conducted on suspected populations. There is a need to integrate omics, molecular biology, and bioinformatics to help demonstrate the genetic diversity of *T. gondii* strains, which would aid in the development of more sensitive and specific diagnostic tools, and to identify a potent antigenic candidate for vaccine development against toxoplasmosis. Further investigation is needed to comprehend the impact of species specificity and the inhibition of delta-6-desaturase activity on the sexual development of *T. gondii* in the intestines of non-feline hosts.

## Figures and Tables

**Table 1:** Milestones in *T. gondii* research in animals.

Year	Finding	Ref.
History		
1909	Name “*Toxoplasma gondii*” proposed	[20]
1937	Successful isolation of viable *T. gondii*	[10]
1992	*T. gondii* was classified as Type I, II, III, and atypical	[12]
2005	Gene mapping achieved	[12,21]
Parasite Morphology and Life Cycle		
1928	Cyst recognized	[4]
1958	Tachyzoites division by endodyogeny described	[22]
1960	Bradyzoites resistance to digestive enzymes recognized	[19]
1970	Oocyst described	[23]
1970	Asexual and sexual stages were reported in the intestine of cats	[24]
1972	Asexual enteroepithelial stages were designated as types A–E	[24]
1973	The term “bradyzoite”, also called cystozoites, was proposed for the cystic organisms	[25]
1973	Term tachyzoite proposed (tachy = fast, zoite = life)	[25]
1988	Term tissue cyst proposed	[12]
2019	*T. gondii* sexual reproduction is associated with delta-6-desaturase gene	[26]
2023	ROCY1 gene activates *T. gondii* bradyzoite formation	[13]
Transmission		
1959	Congenital transmission was observed in mice for up to 10 generations	[27]
1954	Transmission by carnivorism explained	[28]
1965	*T. gondii* infectivity is associated with cat feces	[29]
1970– 1972	Sexual life cycle to defined only in felines, including excretion of oocysts only by felids	[12]
2008	Congenital transmission found in a large wild animal species, the white-tailed deer	[12]
Diagnosis		
1958	Sabin–Feldman dye test	[30]
1968	IgM antibody detection from cord blood or infant serum for detecting congenital toxoplasmosis proposed	[12]
1965	Direct agglutination test (DAT) in humans and other animals	[12]
1969	Detection of IgM in cord blood through the indirect fluorescent antibody test and ELISA	[12]
1989	Detection of *T. gondii* DNA was achieved, using the *B1* gene from tachyzoite, through PCR	[12]
Treatment		
1942	Sulfonamides were reported to be effective against murine toxoplasmosis	[31,32]
1953	Combined therapy with sulfonamides and pyrimethamine was discovered to have satisfactory results in treating toxoplasmosis in humans	[31]
1958	Spiramycin found to have anti-*T. gondii* activity in mice	[33]
1973	Clindamycin was documented to have anti-*T. gondii* effects	[34,35]
Prevention and Control		
1972	Susceptible populations should avoid contact with oocysts	[12]
1990s	Keeping cats out of pig farms can reduce *T. gondii* infection in pigs	[12]
1995	Vaccination of sheep to reduce neonatal mortality in lambs is made available commercially	[36]
First Confirmed Cases of Toxoplasmosis in Animals		
1908	First report of *T. gondii* (bradyzoites) in tissue (cyst) of *Ctenodactylus gundi*, in Tunisia	[1]
1914	Eurasian red squirrels (*Sciurus vulgaris*)	[37]
1916	Howler monkey (*Alouatta seinculus*)	[38]
1951	Squirrel monkeys (*Saimiri sciureus*) in the Philadelphia Zoo	[38]
1974	Pallas’s cats (*Otocolobus manul*)	[39]
1985	Wild turkeys from Blairsville, Georgia, USA	[40]
1987	Bobcat (*Lynx rufus*) from Ronan, Montana, USA	[41]
1990	Koalas (*Phascolarctos cinereus*); an arboreal marsupial, Sydney, Australia	[42]
1990	Dolphins (*Stenella longirostris*)	[43]
1995	Common mole (*Talpa europaea*) from Germany	[44]
1997	Golden lion tamarins (*Leontopithecus rosalia*)	[45]
1997	Barred owl (*Strix varia*) from Quebec, Canada	[46]
2000	Sea otters (*Enhydra lutris nereis*)	[47]
2000	Hawaiian crow (*Corvus hawaiiensis*)	[48]
2004	Striped skunk (*Mephitis mephitis*); *T. gondii* genotype III from Mississippi, USA	[49]
2004	Beaver (*Castor canadensis*)	[50]
2004	Captive nilgais (*Boselaphus tragocamelus*)	[51]
2004	Saiga antelope (*Saiga tatarica*)	[51]
2004	Bald eagle (*Haliaeetus leucocephalus*) from New Hampshire	[52]
2004	Canada goose (*Branta canadensis*); *T. gondii* genotype III from Mississippi, USA	[49]
2004	Black-winged lory (*Eos cyanogenia*); *T. gondii* genotype III from South Carolina, USA	[49]
2005	Hawaiian monk seal	[53]
2005	Fisher (*Martes pennanti*) from Garrett County, Maryland, USA	[54]
2007	Cheetah (*Acinonyx jubatus*) from Dubai, UAE	[55]
2007	American Red squirrels (*Tamiasciurus hudsonicus*) from New York, USA	[56]
2007	Woodchuck (*Marmota monax*) from New York, USA	[56]
2008	Sand cats (*F. margarita*) from Sharjah, UAE	[57]
2008	Gordon’s wildcat (*Felis silvestris gordoni*) from Sharjah, UAE	[58]
2008	Nicobar pigeons from South Africa	[59]
2011	Red-handed howler monkey (*Alouatta belzebul*)	[60]
2011	Black-footed penguin (*Spheniscus demersus*)	[61]
2012	Flying-foxes (megachiropteran bats)	[62]
2013	Northern shoveller (*Anas clypeata*) from Tuscany, Italy	[63]
2013	Common teal (*Anas crecca*) from Tuscany, Italy	[63]
2015	Giant panda (*Ailuropoda melanoleuca*) from Zhengzhou, China	[64]
2015	Wombats (*Vombatus ursinus*)	[65]
2016	Swinhoe’s striped squirrel (*Tamiops swinhoei*) from Germany	[66]
2016	Opossums (*Didelphis virginiana*) from Yucatan, Mexico	[67]
2019	Eastern fox squirrels (*Sciurus niger*)	[68]
Domestic Animals		
Pet animals		
1908	Domestic rabbits (*Oryctolagus cuniculus*) from Brazil	[1,20]
1932	Ferrets (*Mustela putorius furo*) from New Zealand	[69]
1942	Cats from the Middletown, New York, USA	[12]
1950	Dogs	[70]
1956	Mink	[71]
1985	Red foxes (*Vulpes vulpes*)	[72]
Production Animals		
1952	Swine	[73]
1957	Sheep	[74]
1961	Chickens	[75]
1979	Goats	[76]
1990	Camels	[77]
2003	Ducks	[78]
2014	Alpaca	[79]
2014	Turkeys	[80]
2014	Geese from Hainan, China	[81]
2021	Horses	[17,18]
2024	Buffalos	[82]
2025	Cattle	[16]

**Table 2:** Notable outbreaks/epizootics in domestic and wild species.

Year	Animal Specie		Affected/Total	Region	Ref
1952	Chickens		High mortality (flock eradicated)	South-Eastern Norway	[103]
1992	Canaries		7/30 with ophthalmic lesions	New Zealand	[101]
2004	Lories		5/5 died	Columbia, South Carolina, USA	[49]
2008	Nicobar pigeons		3/10 died	South Africa	[59]
2011	Black-footed penguins		3 chicks died	Netherlands	[61]
2019	Chickens and guinea fowl		15/76 died	Viamão, Rio Grande do Sul, Southern Brazil	[102]
1973	Goats		3 does congenital toxoplasmosis (Flock 1)	Tasmania, Australia	[76]
1977			5 stillbirths (Flock 2)		
1978			An abortion having twin kids (Flock 3)		
1954	Sheep		15–20% abortion rate	Wellington, New Zealand	[14,15]
2005			38 abortions + 15 resorbed	Texas, USA	[83]
2006			2 of 26 infected lambs	Texas, USA	[83]
2010			30/239 abortions in a dairy flock	Palencia, Spain	[86]
			70/210 abortions in a meat flock	Segovia, Spain	
2010			40/100 pregnant ewes aborted	Serro, Minas Gerais State, Brazil	[85]
2015– 2018			146/242 from 11 abortion episodes	Spain	[84]
			213/342 from two slaugheterhouses		
1999	monkeys	Wooly monkeys	3 died	Brazil	[98]
2001 and 2006		Squirrel monkeys (*Saimiri sciureus*)	50 monkeys died and none recovered spontaneously	French Guiana	[99]
2018			7 of 13 dead	Seoul, South Korea	[105]
2019			4 died	Hokkaido, Japan	[106]
2020		Howler monkeys (*Alouatta* sp.)	7 died	Brazil	[100]
1994	Pigs		50–60% morbidity, 10–42% mortality 200/800 pigs died	Mantova, Lombardia, Italy	[88]
			810/2080 pigs died	Modena, Emilia-Romagna, Italy	
			~31/345 pigs died		
			34/80 pigs died	Mantova, Lombardia, Italy	
2001			42% affected, 8% mortality	China	[88]
2001			33% affected, 2% mortality	China	[5]
2004			19/260 sows died	Jinchang, Gansu Province, China	[87]
Marine					
2001– 2015	Hawaiian Monk Seals (*Neomonachus Schauinslandi*)		8/183 confirmed *T. gondii*	Hawaii, USA	[91]
2009	Bottlenose dolphins		2 died (all seropositive)	Canada	[90]
2013	Hector’s dolphins		7/28 died (25%)	New Zealand	[89]
2015–2019	Northern sea otters (*Enhydra lutris kenyoni*)		22/44 infected	Washington, USA	[96]
Miscellaneous					
1957	Hares (*Lepus timidus ainu*)		8/13 died	Sapporo, Japan	[104]
1964	Chinchillas		44/56 died + 4 abortions	Not specified	[92]
1992	Black-footed ferrets (*Mustela nigripes*)		8 acute deaths + 13 chronic	Kentucky, USA	[93]
1999	Mink		10,408 kits died	Wisconsin, USA	[97]
2006–2010	Tammar wallabies		Deaths; 6 confirmed + 11 suspected	Budapest, Hungary	[94]
2017	Red-necked wallabies		9 died	Virginia, USA	[95]

**Table 3:** Milestones in *T. gondii* research in humans.

Year	Finding	Ref.
1939	First isolate of *T. gondii* from human.	[11,12]
1939	First report of congenital transmission demonstrated in human.	[11]
1940	*T. gondii* isolated in heart, spleen, and other tissues of humans.	[12]
1941	Report of acquired toxoplasmosis, whose isolate became the famous RH strain.	[12]
1941	*T. gondii* isolated from blood of humans.	[12]
1953	Combined therapy with sulfonamides and pyrimethamine was discovered to have satisfactory results in treating toxoplasmosis in humans.	[31]
1958	Spiramycin has been used prophylactically in pregnant women.	[33]
1960	Transmission by carnivorism reported.	[11]
1965	Feco-oral transmission reported.	[11]
1972	Susceptible populations should avoid contact with oocysts.	[12]
1974	(1) An infection acquired during early pregnancy is more damaging to the fetus.	[12]
	(2) Not all women who acquired the infection transmitted it to the fetus.	
	(3) Women seropositive before pregnancy did not transmit infection to the fetus.	
	(4) Treatment with spiramycin reduced congenital transmission, but not clinical disease in infants.	
1976	The RH strain has lost the capacity to produce oocysts in cats.	[12]
1979	The first human toxoplasmosis outbreak described, was through oocyst inhalation/ingestion.	[12]
1983	Reported fatal acute toxoplasmosis-induced encephalitis, almost all of whom were HIV infected individuals.	[12]
1995	Canadian waterborne outbreak of toxoplasmosis.	[12]
2006	*T. gondii* outbreak in Brazil.	[12]

**Table 10:** Summary of molecular methods for detection of *T. gondii* infection.

Molecular Methods	Purpose	DNA Target Regions	Ref.
Conventional PCR	Species confirmation	*B1* gene, 529-bp repetitive element, *18S rDNA* gene, *SAG1*, *SAG2*, and *GRA1*	[268,269]
Real-time PCR		*B1* gene, 529-bp repetitive element, *18S rDNA* gene, *SAG1*	
LAMP		529-bp repetitive element, *B1*, *SAG1*, *SAG2*, *GRA1*, oocyst wall protein genes	[270]
Microsatellite analysis	Genotyping	*TUB2*, *W35*, *TgM-A*, *B18*, *B1*7; *M33*, *IV.1*, *XI.1*, *M48*, *M102*, *N60*, *N82*, *AA*, *N61*, and *N83*	[271]
Multilocus sequence typing		*BTUB*, *SAG2*, *GRA6*, and *SAG3*	[272]
PCR-RFLP		*SAG1*, *SAG2*, *SAG3*, *BTUB*, *GRA6*, *c22-8*, *c29-2*, *L358*, *PK1* and Apico	[272,273]
RAPD-PCR		Genomic DNA	
High-resolution melting (HRM) analysis		*B1* gene	[274]

**Table 11:** Drugs used to treat animal toxoplasmosis.

Sr.	Drugs	Dose *	Specie	Ref.
1	Sulfadiazine	15–25 mg/kg	all animals	[278,279]
	Pyrimethamine	0.44 mg/kg		[280]
2	Trimethoprim-sulfamethoxazole	15 mg/kg	dogs and cats	[279]
3	Clindamycin	10–12.5 mg/kg	dogs	[281]
		25–50 mg/kg	cats	[127]

* PO: Administered orally, every 12 h for 4 weeks.

**Table 12:** Types of proposed vaccines used against toxoplasmosis.

Sr.	Vaccine Type	Description	Advantages	Limitation	Ref.
1	ESA vaccines	Excretory–secretory antigens (ESAs) are used as antigenic agents	Reduces parasitemia of highly virulent strains. Propranolol and alum-adjuvanted ESE vaccine improve the protective effect of ESA and extend the survival time of mice.	Does not protect against all strains	[293]
2	Live attenuated vaccines e.g., modified *T. gondii* strain (*S48*) vaccine *ΔHAP2* parasites by CRISPR/Cas9 *ΔCDPK2* and *ΔADSL* knock-out vaccine	*T. gondii* is attenuated through gamma radiation, chemical treatment and passages.	Currently, live attenuated vaccine is the most effective anti-*T. gondii* vaccine. If oral live attenuated vaccines are prepared, they can mimic natural *T. gondii* infection and induce host cellular and humoral immunity against *T. gondii* without causing disease.	Short shelf life, may revert to virulence	[294]
3	Subunit vaccines e.g., *rTgHSP70* subunit vaccine *rROP18* and *rCDPK6* combined with *PLG* subunit vaccine	Immunogenic parts of *T. Gondii.*	Multi-epitope subunit vaccines with different T-cell and B-cell epitopes are more promising for research.	Poor immunogenicity, requires carrier	[298]
4	DNA vaccines e.g., *ROP*, *GRA*, *MIC*, and *SAG* antigen vaccines	Made by antigenic proteins such as rhoptry proteins (*ROPs*), microsomal proteins (*MICs*), and surface antigens (*SAGs*).	Inexpensive, easy to administer, and induces a strong immune response.	Poor immunogenicity in large animals, may trigger antibodies against the DNA vector	[137,298]
5	Multiple epitope vaccines SAPNs vaccine and SAPNs scaffolded peptide epitopes vaccines	Antigenic peptide epitopes from *SAG1*, *SAG2*C, *GRA6*, *GRA5* are used as antigenic agents.	Generates stronger Th1 responses and allows epitope optimization.	Poor immunogenicity, short half-life, may cause immune tolerance	[300]
6	mRNA vaccines e.g., *TgNTPase-II*-LNP vaccine	mRNA-stimulating antigenic protein.	Safe, no risk of gene recombination.	Short intracellular half-life, not very stable in vivo	[301]
7	Carbohydrate-based vaccines	*T. gondii* proteins tagged with surface carbohydrates to stabilize and transport them, eliciting carbohydrate-specific antibodies.	*TLR-2* and *TLR-4* can recognize *T. gondii* GPI.	Poor immunogenicity, autoimmunity	[302]
8	Exosome vaccines e.g., DC2.4 exosome vaccine	Exosomes from *T. gondii*, and DC2.4 cell-derived exosomes as antigenic agents.	Induce humoral and cell-mediated immunity.	Poor biocompatibility	[303]

## Data Availability

Not applicable.
